# Supplementary material for: The Association between Sleep and Chronic Spinal Pain: A Systematic Review from the Last Decade
Source: J Clin Med. 2021 Aug 26;10(17):3836. doi: 10.3390/jcm10173836 (PMC8432009; doi:10.3390/jcm10173836)
Supplement: Supplementary file 1 [file jcm-10-03836-s001.zip › jcm-1342282-supplementary.pdf]

Table S1. Search Strategy.

**Embase**<br><br>(((chronic OR persistent OR lasting OR longterm OR 'long term') AND (('backache'/exp OR 'backache' OR 'back ache' OR 'back pain' OR 'backpain' OR 'dorsalgia' OR 'pain, back' OR 'lumbago' OR 'lumbal pain' OR 'lumbalgesia' OR 'lumbalgia' OR 'lumbar pain' OR 'lumbodynia' OR 'lumbosacral pain' OR 'pain, lumbosacral' OR 'pain, low back' OR 'lowback pain') OR ('spinal pain'/exp OR 'spinal pain' OR 'rachialgia' OR 'pain, spinal' OR 'vertebral pain') OR ('whiplash injury'/exp OR 'whiplash injury' OR 'whiplash associated disorder'/exp OR 'whiplash associated disorder' OR 'whiplash') OR ('failed back surgery syndrome'/exp OR 'failed back surgery syndrome' OR 'fbss' OR 'failed back surgery' OR 'failed back syndrome' OR 'post-laminectomy syndrome' OR 'postlaminectomy syndrome') OR ('cervicobrachial neuralgia'/exp OR 'arm neck shoulder syndrome' OR 'cervical brachial neuralgia' OR 'cervical compression syndrome' OR 'cervical radiculopathy' OR 'cervical syndrome' OR 'cervico-brachial neuralgia' OR 'cervicobrachial disease' OR 'cervicobrachial neuralgia' OR 'cervicobrachial pain' OR 'cervicobrachial syndrome' OR 'cervicobrachialgia' OR 'cervicobrachialgic syndrome' OR 'neck shoulder arm syndrome' OR 'neuralgia, cervicobrachial' OR 'perispondylitis cervicalis rheumatica' OR 'radiculopathy, cervical' OR 'shoulder arm neck syndrome') OR ('neck pain'/exp OR 'neck pain' OR 'pain, neck' OR 'cervical pain'))) AND ('sleep'/exp OR 'insomnia'/exp OR 'sleep quality'/exp OR 'sleep quality' OR 'sleep quantity'/exp OR 'sleep quantity' OR 'sleep deprivation'/exp OR 'sleep deprivation' OR 'sleep fragmentation'/exp OR 'sleep fragmentation' OR 'polysomnography'/exp OR 'polysomnography' OR 'polysomnograph'/exp OR 'polysomnograph' OR 'sleep*' OR 'insomni*' OR 'agrypnia' OR 'hyposomnia' OR 'polysomnograph*' OR 'somnography, poly')) AND ([dutch]/lim OR [english]/lim) AND [humans]/lim AND [2009-2020]/py
**PsycARTICLES**<br><br>((((chronic OR persistent OR lasting OR longterm) AND (neck OR back OR spinal) AND (pain OR ache)) OR whiplash) AND (sleep OR insomnia OR polysomnography)) Filters activated: humans, English
**Web of Science**<br><br>(TS=(((("Back Pain" OR "Low Back Pain" OR "Neck Pain" OR "Whiplash Injuries" OR "Failed Back Surgery Syndrome" OR "Brachial Plexus Neuritis" OR backache OR backpain OR dorsalgia OR lumbago OR lumbalgia OR lumbodynia OR whiplash OR fbss OR "failed back surgery" OR "failed back syndrome" OR "postlaminectomy syndrome" OR "post-laminectomy syndrome" OR ((back OR neck OR cervical OR spinal OR vertebral OR lumbar OR lumbosacral) AND (ache OR pain)) OR ((cervicobrachial* OR cervico-brachial OR cervical) AND (neuralgia OR pain OR syndrome OR disease OR radiculopathy OR "compression syndrome")) OR "neck shoulder arm syndrome" OR "shoulder arm neck syndrome")) AND (persistent OR chronic OR lasting OR longterm OR "long term")) AND (sleep OR sleeping OR (sleep initiation and maintenance disorders) OR insomni* OR agrypnia OR (sleep deprivation) OR polysomnography))) AND LANGUAGE: (English)
**PubMed**<br><br>(((("Back Pain"[Mesh] OR "Low Back Pain"[Mesh] OR "Neck Pain"[Mesh] OR "Whiplash Injuries"[Mesh] OR "Failed Back Surgery Syndrome"[Mesh] OR "Brachial Plexus Neuritis"[Mesh] OR backache OR backpain OR dorsalgia OR lumbago OR lumbalgia OR lumbodynia OR whiplash OR fbss OR "failed back surgery" OR "failed back syndrome" OR "postlaminectomy syndrome" OR "post-laminectomy syndrome" OR ((back OR neck OR cervical OR spinal OR vertebral OR lumbar OR lumbosacral) AND (ache OR pain)) OR ((cervicobrachial* OR cervico-brachial OR cervical) AND (neuralgia OR pain OR syndrome OR disease OR radiculopathy OR "compression syndrome")) OR "neck shoulder arm syndrome" OR "shoulder arm neck syndrome")) AND (persistent OR chronic OR lasting OR longterm OR "long term")) AND (sleep[MeSH Terms] OR sleep OR sleeping OR (sleep initiation and maintenance disorders[MeSH Terms]) OR insomni* OR agrypnia OR (sleep deprivation[MeSH Terms]) OR polysomnography[MeSH Terms] OR polysomnography))<br>Filters activated: Publication date from 2009/01/01 to 2020/06/01, Humans, Dutch, English.
